# The influence of fears of perceived legal consequences on general practitioners’ practice in relation to defensive medicine – a cross-sectional survey in Germany

**DOI:** 10.1186/s12875-024-02267-x

**Published:** 2024-01-12

**Authors:** Katja Goetz, Dorothee Oldenburg, Christina Jana Strobel, Jost Steinhäuser

**Affiliations:** Institute of Family Medicine, University Medical Center Schleswig-Holstein, Campus Lübeck, Ratzeburger Allee 160, 23538 Lübeck, Germany

**Keywords:** Defensive medicine, Fear, General practitioner, Legal issues, Cross-sectional study

## Abstract

**Background:**

Medical decisions are influenced by a variety of factors also by legal requirements and feelings of uncertainty, which results in the term defensive medicine. The aim of the study was to evaluate the influence of fears of perceived legal consequences on the practice of defensive medicine from the perspective of German general practitioners (GPs).

**Methods:**

A cross-sectional study was performed from April to May 2022. GPs were invited via an e-mail newsletter of the Institute for Continuing Education in Family Medicine in the German Association of General Practitioners and via an online platform of the German College of General Practitioners and Family Physicians. The evaluation of legal fears, the general assessment of defensive medicine and reasons for and the frequency of defensive medical measures were surveyed in this study. Beside descriptive analyses, a stepwise linear regression analysis was used to explore potential associations between for the primary outcome variable ‘fears of legal consequences’ on the practice of defensive medicine.

**Results:**

413 general practitioners with an average age of 50 years (51% female) responded. The majority rated their fears of legal consequences as low to average whereas for almost a third (27%, *n* = 113) the fears were strong to very strong. Regarding legal fears, the physician-patient-relationship played a fairly to very large role for 48% (*n* = 198) of the respondents. One third estimated the probability of being sued civilly in the next 10 years as rather high to very high. 47% (*n* = 193) of the participants assumed that the risk of being sued could mostly to very much be reduced by defensive medicine. Legal self-protection was for 38% of the responders (*n* = 157) quite frequently to very frequently a reason for acting defensively. Consequently, half of the respondents stated that they performed unnecessary laboratory tests at least once per week and 40% indicated that they referred patients for radiological diagnostics without medical indication once per month.

**Conclusions:**

As legal fears have an influence on medical practice and legal self-protection being a frequent reason for defensive behaviour, understanding and knowledge of the law should be improved by legal education at university and further training of post-graduate trainees and practicing physicians should be implemented. Additionally, a more in-depth enlightenment of society about the phenomenon of Protective and Defensive Medicine and its consequences could be a possibility to decrease the perceived fears of legal consequences on the physicians’ side.

**Supplementary Information:**

The online version contains supplementary material available at 10.1186/s12875-024-02267-x.

## Background

Medical decisions are influenced by a variety of factors such as time pressure and economic constraints and legal requirements [1]. Moreover, medical practice is caught between medical skills as well as legal permissions and obligations. This leads to the feeling that the “Damocles sword of litigation” hovers over the physicians [2]. Thus, uncertainty and fears of legal consequences can result in so-called “defensive medical measures” [3]. Defensive Medicine (DM) is defined as a medical practice in which unnecessary, medically unindicated measures are performed in order to protect oneself from legal consequences and complaints [4]. This so-called “hedging-type” of DM comprises diagnostic examinations without indication, overdiagnosis and overtreatment, such as additional blood tests, referrals, etc [5]. Another type of DM is an avoidance behaviour being a practice in which patients with risky treatments are refused or referred due to fears of legal consequences. It can also be referred to as negative DM [6]. This study was conducted using the hedging-type definition of DM. Related to medical overuse, investigations of DM practice are already focused on in several settings [7, 8]. However, studies investigating the influence of legal requirements and fears in primary care are seldom [9]. The presented study was performed in Germany. Germany is based on a Social Security Health system and is funded by means of earmarked premiums. Patients can chose their medical practitioners freely, the mandatory health insurance pays for the consultation and general practitioners (GPs) are payed by the Association of Statutory Health Insurance Physicians [10]. There is no public patient insurance, which covers medical malpractice injuries. Each GP has a medical liability insurance. Civil liability is usually at the forefront of medical malpractice cases [11].

The aim of this study was to evaluate the influence of fears of perceived legal consequences on GPs actions and the phenomenon of defensive medical practice in general medicine in Germany.

## Methods

### Study design

A cross-sectional study was performed. The study was conducted in Germany and complied with the STROBE guidelines (Strengthening the Reporting of Observational Studies in Epidemiology) [12] (Additional file 1).

### Measurement

The development of the questionnaire was based on the findings of our scoping review of literature regarding factors that influence DM-based decision-making in primary care, particularly, on results of quantitative studies [13]. The pilot-test of the questionnaire regarding its clearness and understandability was performed with one GP, two post-graduate GP trainees and one medical student. Proposals for changes in the wording were incorporated into the final version of the questionnaire. The final questionnaire consisted of three parts: (1) Description of legal fears and requirements on GPs’ practice, (2) General assessment of defensive medicine, (3) Defensive medical practice (reasons and frequency). The software SurveyMonkey was used for the online survey. The full questionnaire is added as Additional file 2.

### Participants and recruitment

Different approaches were used for the recruitment of GPs. The term “GP” comprises general internal medicine physicians and general practitioners in Germany. On April 8th 2022, GPs were invited to participate in the survey through the email newsletter of the Institute for Continuing Education in Family Medicine in the German Association of General Practitioners. The distribution list included 18,500 GPs. One reminder was sent three weeks later. On April 12th 2022, an online invitation to the survey was sent via an online platform of the German College of General Practitioners and Family Physicians (DEGAM) with 1,400 enlisted GPs to the platform, which is voluntary. One reminder was sent nine days, another reminder five weeks later. In this respect, a distinction was not made in this study. The data collection period lasted from April 8th until May 31st, 2022. Online participation in the anonymous questionnaire was classified as informed consent.

### Statistical analysis

The data analysis was conducted using SPSS version 27.0 (SPSS Inc., IBM). Continuous data were summarised using means and standard deviations also for different items (1, 2, 6 to 10, 13 and 14). Categorical data were presented as frequency counts and percentages. Moreover, means, standard deviations and 95% confidence intervals for different items like the examination of the factors influencing GPs’ actions, fears of legal consequences and risk of error/lawsuit were reported. Missing data of below 10% was neglected. For the analysis of reasons for defensive medical practice the responses ‘5 – That was quite often a reason’ and ‘6 – That was very often a reason’ of the different items was summarised to one category. Spearman rank correlation was used to find out on which the independent variables individual characteristics, different aspects of defensive medicine, and various factors of requirements on GPs’ practice showed a significant correlation with the dependent variable ‘fears of legal consequences’. Afterwards, based on the dependent variable ‘fears of legal consequences’ a stepwise linear regression analysis was used to explore potential associations. Additionally, the possibility for multicollinearity was considered. The variance inflation factor (VIF) and the value of tolerance were reported for the last step of both regression models. Values for VIF should not be over 5.0 and for tolerance not lower than 0.25 [14]. An alpha level of P < 0.05 was used for tests of statistical significance.

## Results

In total, 413 participants completed the online questionnaire (response rate 2%). The gender distribution within the survey was almost equal between males and females and similar to the total sample of registered GPs by National Association of Statutory Health Insurance Physicians (Table 1). The respondents’ average age was 50 years and 92% (*n* = 378) were trained GPs. Slightly more than half of the participants had their medical practice in an urban area (56%, *n* = 233). Most of the participants had professional experience for five or less than five years (32%, *n* = 132), followed by 20 years and more (26%, *n* = 108). The majority (74%, *n* = 305) worked self-employed.

**Table 1:** Description of the study sample (*n* = 413)

Characteristics		Numbers (%)	Number of registered physicians (%)^#^
**Gender**	Male	209 (50.8)	26,103 (50.3)
	Female	201 (48.9)	25,820 (49.7)
**Professional title**	General practitioner	321 (78.0)	34,363 (66.2)
	general internal medicine physician	53 (13.0)	17,560 (33.8)
	Post-graduate GP trainee	31 (8,0)	n.a.
**Area of work**	Urban	233 (56.4)	n.a.
	Rural	176 (42.6)	n.a.
**Professional experience**	≤ 5 years	132 (32.0)	n.a.
	Between 6 and 10 years	78 (18.9)	n.a.
	Between 11 and 19 years	93 (22.5)	n.a.
	≥ 20 years	108 (26.2)	n.a.
**Employment form**	Self-employed	305 (73.8)	41,180 (79.3)
	Employed	106 (25.7)	9,473 (18.2)
**Age in years**: mean (SD); range		49.8 (10.6); 25–80	55.3
**Fears of legal consequences**: mean (SD)*		3.5 (1.4)	n.a.
**Estimation of the likelihood of being sued in a job-related civil action in the next 10 years**: mean (SD)*		3.0 (1.3)	n.a.

SD: standard deviation; n.a. not available

*range from 1 “very low” to 6 “very strong”

^#^registered by the National Association of Statutory Health Insurance Physicians (31.12.2022)

### Theme 1: description of legal fears and requirements on GPs’ practice

Concerning “fears of legal consequences” as the main variable of this study, the majority (44%, *n* = 180) rated their legal fears as low to average. The proportions of those for whom these fears were not at all to very low noticeable and those for whom these fears were strong to very strong distincted were almost identical (not at all to very low: 28%, *n* = 117, strong to very strong: 27%, *n* = 113). For almost half of the participants (48%, *n* = 198), the physician-patient-relationship played a fairly to very large role regarding the topic ‘fears of legal consequences’ and how to avoid them. Almost half of the GPs (48%, n = 200) felt influenced to a high or very high degree by legal requirements in their medical practice.

Table 2 presents the description of factors influencing GPs’ actions, fears of legal consequences and risk of error and lawsuit. For example, the means ranging from 1 “very low” to 6 “very high” of the perceived fears of legal consequences was 3.50 (SD 1.42) and of the feeling of influence by legal requirements 4.29 (SD 1.25).

**Table 2:** Descriptive analysis of different items such as factors influencing GPs’ actions, fears of legal consequences and risk of error and lawsuit

Description of different items of the questionnaire		Mean (SD)	95% CI
**Factors influencing GPs’ actions**	Fears of legal consequences*	3.50 (1.41)	3.37; 3.64
	Legal requirements*	4.29 (1.25)	4.17; 4.41
**Factors influencing fears of legal consequences**	Estimation of the likelihood of being sued in a job-related civil action in the next 10 years*	3.06 (1.38)	2.92; 3.19
	Already once a conciliation procedure	1.82 (0.39)	1.78; 1.85
	Sued once already	1.84 (0.37)	1.80; 1.88
	Knowledge of a colleague who has been sued	1.34 (0.47)	1.29; 1.39
	Physician-patient relationship*	4.16 (1.53)	4.00; 4.30
	Legal requirements*	4.29 (1.25)	4.17; 4.41
	Legal protection/ safeguard*	3.84 (1.39)	3.69; 3.98
	Pressure from patients*	3.55 (1.37)	3.40; 3.68
	Pressure from relatives*	3.28 (1.41)	3.14; 3.42
	Peer pressure*	1.94 (1.29)	1.80; 2.07
	Gender	1.50 (0.50.)	1.45; 1.55
	Age	49.5 (10.50)	48.45; 0.56
	Professional title	2.98 (0.67)	2.92; 3.05
	Urban or rural	1.43 (0.48)	1.38; 1.48
	Professional experience	2.41 (1.19)	2.29; 2.53
	Self-employed or employed	1.25 (0.43)	1.20; 1.29
**Factor influencing the risk of errors**	Defensive medicine**	3.62 (1.31)	3.49; 3.75
**Factors influencing the risk of lawsuit**	Defensive medicine***	2.68 (1.07)	2.57; 2.78
	Orientation on guidelines*	4.47 (1.29)	4.34; 4.59

SD standard deviation, 95% CI confidence interval

* items range from 1 “very low” to 6 “very high”

** item with a range from 1 “no increase in risk at all” to 6 “very large increase in risk”

*** item with a range from 1 “risk is greatly reduced” to 6 “risk is greatly increased”

### Theme 2: general assessment of defensive medicine

Almost half of the GPs (47%, *n* = 193) assumed that the risk of being sued civilly could mostly to very much be reduced by defensive medicine. Almost two thirds of the participants (63%, *n* = 261) assumed that the risk of being sued civilly could be reduced by following guidelines in most cases (44%, *n* = 181) or in a lot of cases (19%, *n* = 80). Almost all participants (98%, *n* = 405) were of the opinion that defensive medical measures could result in an increase in health care expenditure. A large group (89%, *n* = 367) believed that defensive medial practice could lead to uncertainty on the patients’ side. The consequence “health damage caused to patients” was selected by 76% (*n* = 313), “negative image of physicians” by 59% (*n* = 242). 14% (*n* = 58) named other consequences in the free text field, in particular “waste of time” and “-resources” 4% (*n* = 17), “rising expectations of patients 2% (n = 9), “over-diagnostics and overtreatment” 1.45% (n = 6) and “deterioration of the physician-patient relationship” 1.21% (n = 5).

### Theme 3: defensive medical practice (reasons and frequency)

For a lot of the GPs in our study (38%, *n* = 157) legal self-protection was a quite to very frequent reason for acting defensively, closely followed by the reason “I was worried about overlooking a serious illness” (35%, *n* = 143) and “I did not have the time to discuss with the patient” (34%, *n* = 140). Further details are shown in Table 3.

**Table 3:** Reasons for defensive medical practice (summarised categories: “5 – That was quite often a reason” to “6 – That was very often a reason”)

Reasons for acting defensively	N (%)
I wanted to cover myself legally.	157 (38.0)
I was worried about overlooking a serious illness	143 (34.7)
I did not have the time to discuss with the patient.	140 (33.9)
I wanted to reassure the patient.	133 (32.2)
Pressure from patients	115 (27.8)
Pressure from relatives	92 (22.6)
I was worried that the patient would complain about me (e.g., press, medical association).	76 (18.4)
I was afraid of negative evaluation on internet platforms.	42 (10.2)
The patient had private health insurance.	29 (7.0)
Peer pressure	24 (5.8)

**Fig. 1:**
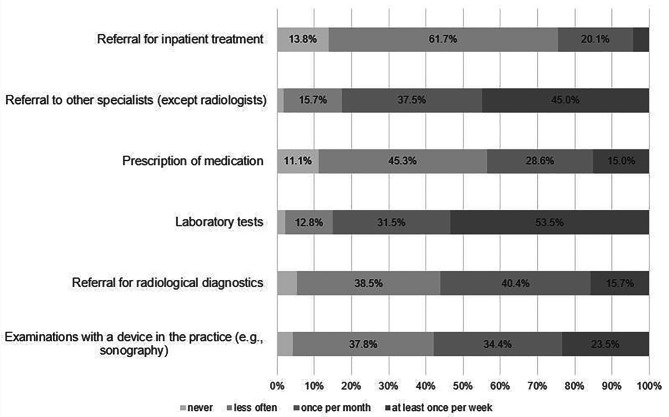
Overview of different defensive medical measures (distribution of percentage)

The following procedures were carried out without medical indication on demand with the frequencies as listed below. At least once per week, 54% (*n* = 221) of the GPs performed unnecessary laboratory tests. Once per month, 40% (*n* = 167) of the participants referred patients for radiological diagnostics unnecessarily. For measures that were carried out less frequently, referrals for inpatient treatment were stated in first place by 62% (*n* = 255). All results are shown in Fig. 1.

5% (*n* = 20) named other defensive measures, 9 of them with a frequency indication. At least once per week: unnecessary sick leaves 0.73% (*n* = 3) and prescriptions for remedies such as physiotherapy 0.73% (*n* = 3). Once per month: control blood pressure measurements, prescriptions for remedies and the use of individual health services which must be paid by patients themselves, once each.

Spearman rank correlation showed significant correlations with different aspects of defensive medicine, and various factors of requirements on GPs’ practice on fears of legal consequences. The items 4, 5, 9, 11, and 13 showed no significant correlation and were excluded from the following regression analysis.

### Associations of individual characteristics, various aspects of defensive medicine on fears of legal consequences

Table 4 shows the stepwise linear regression models for dependent variable ‘fears of legal consequences’ and independent variables (e.g. peer pressure, pressure from patients, referral to other specialists) which correlated significantly with the dependent variable as well as the individual characteristics. A model with seven steps was carried out and explained more than 47% (R² ~ 0.47) of the variance of ‘fears of legal consequences’. In the first step of the stepwise regression analysis, the item ‘I wanted to cover myself legally’ showed the highest score (R² = 0.318). A “higher legal protection of myself” was associated with more fears of legal consequences (ß= 0.566). Moreover, pressure from patients (ß= -0.099) and referral to other specialists (ß= -0.082) was associated with lower fears of legal consequences. The statistics of collinearity for this model ranged between 1.553 (VIF-value), 0.644 (tolerance value) for ‘I wanted to cover myself legally’ and 1.047 (VIF-value), 0.955 (tolerance value) for ‘gender’.

**Table 4:** Associations of individual characteristics, various aspects of defensive medicine on fears of legal consequences (results of stepwise linear regression analysis, under specification of standardized beta coefficient, α = 5%)

	Step 1	Step 2	Step 3	Step 4	Step 5	Step 6	Step 7
I wanted to cover myself legally.	0.566	0.458	0.360	0.333	0.328	0.351	0.338
Likelihood of being sued civilly on an occupational basis in the next 10 years		0.316	0.256	0.257	0.249	0.256	0.250
Feeling of influence of legal requirements on GPs’ actions			0.232	0.266	0.260	0.257	0.249
Gender				− 0.153	− 0.151	− 0.155	− 0.158
Peer pressure					0.076	0.091	0.080
Pressure from patients						− 0.081	− 0.099
Referral to other specialists							− 0.082
**R²**	**0.318**	**0.405**	**0.441**	**0.462**	**0.466**	**0.470**	**0.474**

## Discussion

The aim of this study was to evaluate the influence of fears of perceived legal consequences on GPs’ actions and the phenomenon of defensive medicine in primary care in Germany.

### Influence of legal fears and requirements on GPs’ practice

The largest group of participants rated their fears of legal consequences as low to average, but for almost a third, the fears were strong to very strong. These results are comparable to the results of studies from 1995 to 2014 [15, 16]. For almost half of the GPs, the physician-patient-relationship played a large to very large role regarding the topic “Fears of legal consequences”. We found no data concerning this point in further quantitative surveys. However, studies emphasized the importance of a positive, trusting physician-patient relationship, especially to mitigate the effects of legal fears and defensive medical behaviour [17]. Moreover, a positive physician-patient-relationship was seen as the key to differentiate between „cautious“ medicine and purely defensive medicine [18]. Unfortunately, comparative data regarding the question of how many physicians have already been sued under civil law is not yet available in Germany. In particular, statistics on proceedings settled before German district courts in civil medical malpractice cases do not differentiate between medical specialties. However, we found related data in international literature. According to these between 6% and 25% of the participating GPs stated that they had already been sued for malpractice [16, 19]. Our results of the regression analysis regarding the assessment of the probability of being sued in the next 10 years in a professional civil law case being rather high as well as the need for legal protection. However, no quantitative studies were found in national literature regarding personal knowledge of lawsuits against colleagues working as a GP. According to an international study, one-third of the GPs had been informed of lawsuits against their colleagues [20]. In comparison with this survey, again, the proportion in our study is quite high.

### General assessment of defensive medicine

In line with the results of our study, another study found that half of the GPs thought defensive medicine could decrease the risk of ever being sued [16]. Others mentioned that there is almost no empirical evidence that defensive medical measures will reduce the risk of being sued [19]. In contrast to our result our study, that the risk of litigation could be reduced by adherence to guidelines, there are currently quite few guidelines for general medicine in Germany (e.g., the guideline on multimorbidity) available [21]. However, guidelines might exert pressure and lead to defensive medical actions themselves [22]. Regarding health expenditure, our result roughly corresponds to the result of a member survey of the German Society of Internal Medicine [23]. Another study also dealt with the financial consequences of DM and stated that an increased use of DM may result in increased costs and economic changes to the health care system [15]. In line with our findings, DM can lead to patients being more concerned because something may be discovered that was not even looked for and is unlikely to be relevant [24]. The potential health damage caused to patients and negative image of physicians as consequences of defensive medical measures were also described in other studies [25, 26].

### Defensive medical practice (reasons and frequency)

In the literature, reasons for defensive medicine were mainly seen in avoiding emotional consequences of feelings like guilt or distress if a serious illness has not been detected in time [27, 28]. Furthermore, we found several quantitative studies which had investigated the frequency of reasons for defensive measures. One study depicted patient influence, concern for overlooking severe disease and influence from patient relatives as most frequent reasons for action defensively and only seldomly for concern of patient claims [29]. According to another study patient pressure, anxiety relief, the fear of a legal claim and the fear of negative publicity/image were reasons for defensive actions [30]. The majority of these studies mentioned patient pressure as a frequent reason. In our study it is mentioned in the fifth place. This is in line with our regression analysis that shows that patient pressure plays no relevant role concerning the fears of legal consequences. Further research should address whether GPs feel obligated to comply with the legislation law rather than to comply with patients’ requests. As in our study, most studies mentioned concerns for overlooking a severe illness as a reason for acting defensively. Findings in international literature are in line with our results. For example, studies showed 60–70% of unnecessary testing and in others about one-third to two-third referred patients to other specialists unnecessarily [15, 30, 31].

### Strengths and weaknesses

To our knowledge, the first cross-sectional study dealing with the topic of fears of perceived legal consequences in relation to defensive medicine in primary care in Germany was presented. Male and female participants provided nearly equal representation within the survey, giving confidence to the findings in this regard. The sociodemographics of the participants in the sample were well comparable with those of all GPs in Germany [32]. No response rate could be given since recruitment was done online and it was unclear how many GPs were reached in total. Moreover, the questionnaire did not specify the legal requirements and no distinction were made between DM, which is doctor-centred and Protective Medicine, which protects both the patient and the doctor from the risk of harm. Additional, subjective reporting influenced the perspective of the participants on the survey. Therefore, the risk of a self-selection bias exists, with maybe only those who are already interested in the subject participating in the survey. Finally, this was an exploratory study and thus we must be cautious when deriving causal links from these findings.

## Conclusions

Our study results show that fears of perceived legal consequences have an influence on GPs’ actions in primary care in Germany and that legal self-protection is a frequent reason for defensive medicine. Therefore, teaching of legal issues, e.g., an understanding of key legal principles generally and in medicine law specifically, should be included in medical education and postgraduate training. Moreover, the training needs to be include aspects like why diagnostic uncertainty and errors occur and how these diagnostic risks can be managed safely through protective rather than defensive medicine. It is important is that doctors know how to respond to the threats of complaints and litigation in a way that is appropriate, balanced and protective of both doctor and patient. Additionally, a more in-depth enlightenment of society about the phenomenon of Protective and Defensive Medicine and its consequences could be a possibility to decrease the perceived fears of legal consequences on the physicians’ side. Finally, our findings confirm the importance of effective patient communication skills to reduce legal risks.

### Electronic supplementary material

Below is the link to the electronic supplementary material.

Additional file 1

Additional file 2

## Data Availability

The datasets used and analysed during the current study are available from the corresponding author on reasonable request.

## Abbreviations

CI: Confidence interval
DEGAM: German College of General Practitioners and Family Physicians
DM: Defensive Medicine
GP: General practitioner
SD: Standard deviation
VIF: Variance inflation factor

## References

[1] Schattner A (2009). Angst-driven medicine?. Q J Med.

[2] Dudeja S, Dhirar N (2018). Defensive medicine: Sword of Damocles. Natl Med J India.

[3] Miziara ID, Miziara CSMG (2022). Medical errors, medical negligence and defensive medicine: a narrative review. Clin (Sao Paulo).

[4] Baungaard N, Skovvang PL, Assing Hvidt E, Gerbild H, Kirstine Andersen M, Lykkegaard J (2022). How defensive medicine is defined in European medical literature: a systematic review. BMJ Open.

[5] Ries NM, Jansen J (2021). Physicians’ views and experiences of defensive medicine: an international review of empirical research. Health Policy.

[6] Studdert DM, Mello MM, Sage WM (2005). Defensive medicine among high-risk specialist physicians in a volatile malpractice environment. JAMA.

[7] Lykkegaard J, Andersen MKK, Nexøe J, Hvidt EA (2018). Defensive medicine in primary health care. Scand J Prim Health Care.

[8] Mira JJ, Carrillo I, Silvestre C (2018). Drivers and strategies for avoiding overuse. A cross-sectional study to explore the experience of Spanish primary care providers handling uncertainty and patients´ requests. BMJ Open.

[9] Pellino IM, Pellino G (2015). Consequences of defensive medicine, second victims, and clinical-judicial syndrome on surgeons’ medical practice and on health service. Updates Surg.

[10] Blümel M, Spranger A, Achstetter K, Maresso A, Busse R. Germany. Health system review. WHO. 2020. https://iris.who.int/bitstream/handle/10665/341674/HiT-22-6-2020-eng.pdf?sequence=1 Accessed [01 December 2023]. 34232120

[11] Oldenburg D, Wagner HO, Steinhäuser J (2022). Juristische Implikationen ärztlichen Handelns – Professioneller Umgang Mit Behandlungsfehlern (Legal implications of Physician action – dealing professionally with treatment errors). Z für Allgemeinmedizin.

[12] von Elm E, Altman DG, Egger M, Pocock SJ, Gøtzsche PC, Vandenbroucke JP, STROBE Initiative (2007). The strengthening the reporting of Observational studies in Epidemiology (STROBE) statement: guidelines for reporting observational studies. Lancet.

[13] Strobel CJ, Oldenburg D, Steinhäuser J (2022). Factors influencing defensive medicine-based decision-making in primary care: a scoping review. J Eval Clin Pract.

[14] Field A (2011). Discovering statistics using SPSS.

[15] Summerton N (1995). Positive and negative factors in defensive medicine: a questionnaire study of general practitioners. BMJ.

[16] Brateanu A, Schramm S, Hu B (2014). Quantifying the defensive medicine contribution to primary care costs. J Med Econ.

[17] Assing Hvidt E, Bjørnskov Pedersen L, Lykkegaard J, Møller Pedersen K, Andersen MK (2021). A colonized general practice? A critical habermasian analysis of how general practitioners experience defensive medicine in their everyday working life. Health (London).

[18] Steurer J, Gächter T (2015). Defensive medizin – unnötige Medizin? (defensive medicine – unnecessary medicine?). Swiss Med Forum.

[19] Yüksel O (2021). Evaluation of Family Physicians’ opinions on Defensive Medicine practices: the case of the Province of Isparta/Turkey. Acibadem Univ Saglik Bilim Derg.

[20] Moosazadeh M, Movahednia M, Movahednia N, Amiresmaili M, Aghaei I (2014). Determining the frequency of defensive medicine among general practitioners in Southeast Iran. Int J Health Policy Manag.

[21] Muche-Borowski C, Lühmann D, Schäfer I (2017). Development of a meta-algorithm for guiding primary care encounters for patients with multimorbidity using evidence-based and case-based guideline development methodology. BMJ Open.

[22] Assing Hvidt E, Lykkegaard J, Pedersen LB, Pedersen KM, Munck A, Andersen MK (2017). How is defensive medicine understood and experienced in a primary care setting? A qualitative focus group study among Danish general practitioners. BMJ Open.

[23] Fölsch UR, Faulbaum F, Hasenfuß G (2016). Wie Internisten das Problem Von Über- Und Unterversorgung werten (how internists evaluate the problem of over- and underuse). Dtsch Ärzteblatt.

[24] Bester JC (2020). Defensive practice is indefensible: how defensive medicine runs counter to the ethical and professional obligations of clinicians. Med Health Care Philos.

[25] Vento S, Cainelli F, Vallone A (2018). Defensive medicine: it is time to finally slow down an epidemic. World J Clin Cases.

[26] Catino M (2011). Why do doctors practice defensive medicine? The side-effects of medical litigation. Safe Sci Monit.

[27] Hambrock U. Erfahrungen mit Überversorgung. Qualitativ-psychologische Studie mit Patienten und Ärzten. Bertelsmann-Stiftung, Gütersloh, 2019; https://www.bertelsmann-stiftung.de/en/publications/publication/did/erfahrungen-mit-ueberversorgung-2. Accessed [03 July 2023].

[28] Grote Westrick M, Volbracht E, Deckenbach B, Nolting HD, Zich K. Überversorgung – eine Spurensuche (Overuse - a search for clues). Bertelsmann-Stiftung, Gütersloh, 2019; https://www.bertelsmann-stiftung.de/en/publications/publication/did/ueberversorgung-eine-spurensuche?print=1. Accessed [03 July 2023].

[29] Andersen MK, Hvidt EA, Pedersen KM, Lykkegaard J, Waldorff FB, Munck AP, Pedersen LB (2021). Defensive medicine in Danish general practice. Types of defensive actions and reasons for practicing defensively. Scand J Prim Health Care.

[30] Hasan B, Abdulrahim H, AlMukhtar M, AlAsfoor R, Mandeel M (2018). The practice of Defensive Medicine by doctors in primary health care in the Kingdom of Bahrain. Saudi J Med.

[31] Summerton N (2000). Trends in negative defensive medicine within general practice. Br J Gen Pract.

[32] National Association of Statutory Health Insurance Physicians. 31.12.2022. https://gesundheitsdaten.kbv.de/cms/html/16392.php Accessed [01 December 2023].

