# Supplementary material for: The influence of fears of perceived legal consequences on general practitioners’ practice in relation to defensive medicine – a cross-sectional survey in Germany
Source: BMC Prim Care. 2024 Jan 12;25:23. doi: 10.1186/s12875-024-02267-x (PMC10785451; doi:10.1186/s12875-024-02267-x)
Supplement: Supplementary file 2 — Additional file 2 [file 12875_2024_2267_MOESM2_ESM.pdf]

Additional file 2: Translated_Questionnaire.

„Damocles“-study: To what extent do fears of legal consequences influence actions of general practitioners?

How high – do you think – is the estimated likelihood of being sued in a job-related civil action in the next 10 years?

1. (Estimating the likelihood of being sued in a job-related civil action in the next 10 years)

2. How distinct are your fears of legal consequences?

3. Has a conciliation procedure already been initiated against you?

4. Have you ever been sued civilly by a patient?

5. Do you personally know a GP colleague who has been subject to a civil lawsuit?
   → yes/no

6. To what extent does the doctor-patient relationship play a role for you concerning "fears of legal consequences"? (1 = no role at all, 6 = very great role)

7. To what extent do you feel influenced by legal requirements in your medical practice?

(1 = no feeling of influence at all, 6 = feeling of very great influence)

Defensive medicine is understood to mean, among other things, that unnecessary, i.e., medically unnecessary, additional measures are carried out in order to protect oneself legally.

8. To what extent do you think defensive medicine increases the risk of errors? (1 = no increase in risk at all, 6 = very large increase in risk)

9. To what extent do you think defensive medicine changes the risk that you could be sued civilly? (1 = risk is greatly reduced, 6 = risk is greatly increased)

10. To what extent do you think that following guidelines will reduce the risk that you could be sued civilly? (1 = no risk reduction at all, 6 = very great risk reduction)

11. In your opinion, what are the consequences for our health care system of benefits that serve primarily to provide legal protection? (Multiple answers possible) Yes: No:
    - Increase in health care expenditures,
    - Uncertainty of patients,
    - Health damage caused to patients,
    - Negative image of physicians,
    - Other consequences, namely:

12. How often have you performed the following procedures when urged to do so without medical indication?

at least once per week, once per month, less often, never

- Examinations with a device in the practice (e.g., sonography)
- Referral for radiological diagnostics
- Laboratory tests
- Prescription of medication
- Referral to other specialists (except radiology)
- Referral for inpatient treatment
- Other measure, namely .... with one of the above-mentioned frequencies

13. In your experience, how often was the following the reason you acted defensively? (Multiple answers possible)

    (1 = That was never a reason. 6 = That was very often a reason.)

a) I did not have time to discuss with the patient.

b) I was worried that the patient would complain about me (e.g., press, medical association).

c) I was worried about overlooking a serious illness.

d) The patient had private health insurance.

e) I wanted to reassure the patient.

f) I wanted to cover myself legally.

g) I was afraid of negative evaluation on internet platforms.

h) There was pressure from the patient.

i) There was pressure from relatives.

j) There was pressure from colleagues (e.g., peer pressure).

14. How often do you recommend a guideline to a colleague to protect patients from unnecessary medical procedures? (1 = never, 6 = very often)

15. On which of the following topics would you like further training? Please select a maximum of three topics.

- What does the law actually require? (e.g., medical documentation)
- Basic principles of medical professional law (e.g., advertising contrary to professional ethics)
- Risk communication with patients (e.g., shared decision-making)
- Professional handling of treatment errors (e.g., basic features of medical liability law)
- Sources of information to protect my patients from overtreatment (e.g., overview of materials to which patients who request or demand unnecessary measures can be referred)

… sociodemographics …

23. What aspect, if any, were you missing from this survey?
